# United States Veterinary Medical Association—Officers and Committees, 1892–3

**Published:** 1892-12

**Authors:** 


### UNITED STATES VETERINARY MEDICAL ASSOCIATION.

### *Officers and Committees for 1892–3.*

Thirteenth Annual Meeting of the United States Veterinary Medical Association and the First International Veterinary Congress of America, World's Fair, Chicago, Ill., Sept., 1893.

*President*, W. L. Williams, Lafayette, Indiana.
*Vice-President*, A. W. Clement, 210 St. Paul St., Baltimore, Md.
*Secretary*, W. Horace Hoskins, 12 S. 37th St., Philadelphia Pa.
*Treasurer*, James L. Robertson, 409 9th Ave., New York City.

*International Committee:*—R. S. Huidekoper, Chairman, 311 W. 59th St., New York City; A. Liautard, 141 W. 54th St., New York City; D. E. Salmon, Bureau of Animal Industry Washington, D. C.; A. H. Baker, 145 Michigan Ave., Chicago, Ill.; J. H. Stickney, American Stables, Sudbury St., Boston, Mass.; Olof Schwartzkopff, 637 Cedar St., St. Paul, Minn. *Ex-officio*, W. L. Williams, Lafayette, Ind.; A. W. Clement, 216 St. Paul St., Baltimore, Md.; W. Horace Hoskins, 12 S. 37th St., Philadelphia, Pa.; J. L. Robertson, 409 9th Ave., New York City.

*Comitia Minora:*—R. S. Huidekoper, Chairman, 311 W. 59th St., New York City; A. Liautard, 141 W. 54th St., New York City; R. A. McLean, 14 Nevins St., Brooklyn, N. Y.; S. Stewart, Nebraska City, Neb.; T. J. Turner, Columbia, Mo.; C. E. Hollingsworth, La Salle, Ind.; J. F. Winchester, Lawrence, Mass.

*Intelligence and Education:*—F. H. Osgood, Chairman, 50 Village St, Boston, Mass.; Tait Butler, Agricultural College, Miss.; A. H. Baker, 145 Michigan Ave., Chicago, Ill.; H. P. Eves, 507 W. 9th St., Wilmington, Del.; L. H. Howard, 1440 Washington St., Boston, Mass.

*Finance:*—C. C. Lyford, Chairman, 821 3d Ave., Minneapolis, Minn.; W. B. E. Miller, 527 Penn St., Camden, N. J.

*Committee on Diseases:*—J. F. Winchester, Chairman, Lawrence, Mass.; Wyatt Johnston, 99 Union Ave., Montreal, Canada; Jos. Hughes, 2537 State St., Chicago, Ill.; J. B. Paige, Amherst, Mass.; S. Stewart, Nebraska City, Neb.

*Prize:*—C. P. Lyman, Chairman, 50 Village St., Boston, Mass.; W. H. Lowe, 190 Ellison St., Paterson, N. J.; Tait Butler, Agricultural College, Miss.

*Army Legislation:*—Olof Schwartzkopff, Chairman, 637 Cedar St., St. Paul, Minn.; D. Lemay, Fort Riley, Kansas; C. B. Michener, Dept. of Agriculture, Washington, D. C.

*Publication Committee:*—W. Horace Hoskins, Chairman, 12 S. 37th St., Philadelphia, Pa.; Austin Peters, 23 Court St., Boston, Mass.; T. B. Rayner, Chestnut Hill, Philadelphia, Pa.

*Special Committees:*—To consider the subjects and to outline the scope of work for our International Meeting.

*Veterinary Education:*—A. Liautard, Chairman, 141 W. 54th St., New York City; M. Stalker, Ames, Iowa; C. P. Lyman, 50 Village St., Boston, Mass.

*Tuberculosis Committee:*—A. W. Clement, Chairman, 210 St. Paul St., Baltimore, Md.; Leonard Pearson, 2200 Pine St., Phila., Pa.: Austin Peters, 23 Court St., Boston, Mass.

*Animal Food:*—D. E. Salmon, Chairman, Bureau of Animal Industry, Washington, D. C.; Olof Schwartzkopff, 637 Cedar St., St. Paul, Minn.

*Honorary Members:*—R. S. Huidekoper, Chairman, 311 W. 59th St., New York City; A. Liautard, 141 W. 54th St., New York City; Leonard Pearson, 2200 Pine St., Phila., Pa.

